# Our Dear Ones and Ourselves

**Published:** 1919-11-01

**Authors:** 


### THE WORST WAR OF THE WORLD.

### XI. OUR DEAR ONES AND OURSELVES.

### All Souls' and All Saints' Days Uniting Power Through the Spirit.

All Saints' Day falls this year on the day of publication of the present issue, November 1, 1919. Many of the services held at St. Paul's Cathedral and elsewhere during the war have been faithfully and fully recorded in our columns. Services of Intercession, Services of Submission to God's Will and of Commitment of the souls and bodies of our countrymen and Allies to the care and keeping of God the Father, the purification of Jesus Christ and the sanctification of the Holy Spirit. Ernest and purposeful were the prayers and worship offered at these services, and wonderful indeed has been the response to our prayers and the deliverance which has been granted to the Allies, and especially to the people of the United Kingdom and Empire. The full report of each service and its circulation in our columns throughout the English-speaking world have brought us letters of gratitude and appreciation from many countries and people within the Empire and the United States of America. Attendance at those services brought invaluable fruit and soul-comfort to the majority of those who were privileged to attend them, who carried away with them into the outer world an added strength derived from the recognition of the reality of God's dealing with men and women, of the Eternal Life which flows in such abundance through the sacrifice and death of our Lord and Saviour, and the abiding truth and force of good Bishop Phillips Brooks' immortal prayer: "Oh God, of Thy great goodness keep me alive in Thee through this dying life, that out of death Thou mayest raise us up to immortality, for His sake Who is the Life, Jesus Christ, our Lord. Amen."

The recollection and recall of the wonderful services at St. Paul's Cathedral, in Westminster Abbey and elsewhere cannot fail to fill the mind with many memories, uniting us in the closest ties to the men who offered their lives up in the war for the protection and safety of their country, the Empire and those who were nearest and dearest to them in the same circle. As the years of the war accumulated and passed, facts came out which made every thinking man and woman realise what extraordinary and wonderful courage and heroism were displayed by the small army which constituted the seven divisions which first went forth to war for England and all that she meant to civilised humanity. These divisions came through the retreat from Mons, with one flank exposed, and who yet bore themselves so manfully and bravely that after many days they were able to halt, unite, and, by what can properly be regarded as the Providence of Almighty God, to take a most valiant part in beating the Germans thoroughly, despite all our warriors' sufferings and the strain upon their endurance. They thus placed the first real check on the German intentions to destroy utterly and overcome completely for the sole purpose of promoting their own selfish and brutal ends, and proved indisputably that it must and would fail. Still for many weary months and even years our brave home defenders, reinforced by the Magnificent and Robust warriors who came to the aid of England and her Allies from All Parts of the Empire, and many parts of the world, had to bear the assault of foes ever increasing in number and in implements of destruction.

At length England was awake and all her people were purposefully engaged in the gigantic task of providing war material of a character and amount sufficient to overcome, conquer, and finally to destroy utterly, the enemies whose brutal, inhuman and outrageous proceedings, trickeries, untruths, and disregard of everything that was noble and just between man and man, had made them a byword amongst the nations. Then came the Armistice, followed by a multitude of tangles to be straightened out and huge difficulties in constituting and enforcing the bases of a Peace, broad enough and ample enough to afford the necessary guarantees that the signing of peace would render a World Peace not only possible but lasting and strong. Nor was this all, for the President of the United States of America, Mr. Woodrow Wilson, with magnificent, untiring and wise persistence, put forward and carried the organisation of the League of Nations, which has been accepted and ratified already by a majority of the Allied Powers. The United States of America, for political or other reasons, occupies as we write anything but an enviable position before the civilised nations of the world, owing to the proceedings in and threatened disasters through the United States Senate, where some party politics and ambitions seem willing to wreck the whole world, at any rate so far as its high ideals are concerned, though they originated with the President of the United States of America, and were welcomed by a large majority of the American people. It will of course be disastrous should such an overwhelming calamity be forced upon civilised humanity by a small majority of politicians who may all disappear and pass into the limbo of forgotten things within a few months of the time that their reckless and wanton policy of destruction was forced forward, regardless of the fact that its success might prove a Fatal Blow to The Peace of the World and the reduction of all future war to proportions so small as to render its possibility infinitely insignificant, though not altogether impossible.

☛ Previous articles appeared on July 28, Aug. 11 and 18, Sept. 1 and Dec. 29, 1917, Aug. 10, Nov. 16, Dec. 28, 1918; June 21 and 28, 1919, page 321.

**The Worst War of the World**—*(continued)*.

We have ventured to inflict this terse and rapid summary of the progress, development, ultimate results, and some striking happenings through the years of the Great War. There remains to recall to the remembrance the awful cost in human life of this terrible war and all it has meant to Parents, Wives, Mothers, Families, Nations and Peoples, especially in the Homeland where our men and women gave themselves up with patriotic and earnest completeness, regardless of their own lives and welfare, to the task of securing Victory and Peace. There are few families which do not contain one or more vacant chairs, recalling the dear ones who have Voluntarily and Purposefully Made the Supreme Sacrifice, that England might be secure and the Empire united into a great Kingdom, stronger and better in all respects than was thought to be possible before the war enveloped it. Those of us whose privilege it has been to do our part in the trenches, on the seas, amongst the "U" boats, in foreign lands, wherever duty and war have carried us, will ever remember and thank God for the exhibition everywhere of the whole-hearted self-sacrifice and the yielding up without stint of everything each could supply to bring continuous and increasing force to the work, whatever its character, at the supreme point of danger or in the humblest backwater, wherever duty called and need arose. Splendid indeed was the evidence afforded everywhere of the true and overmastering power of this lofty self-sacrifice by great nations and the whole Empire, filled with men of a fighting race and supported by the denizens of the Far East, and finally by the great centre of progressive industry and invention across the Atlantic. We can testify to the wonder of this evidence, and to the magnificent results which have accrued in the development of science, the saving of life, the awakening, extension and improvement of human effort, the overpowering of every symptom of weakness, and the production of a confidence resting on the belief in God the Father and His ever-watchful Providence, Who stands for justice and right and the ultimate annihilation of everything opposed to its triumph and enforcement.

So, bearing all these things in mind, with grateful hearts, proud as individuals and families of the brave men and women whom we have lost, and who have now been Raised to Immortality, we approach and look forward with full hearts and confidence to the coming of All Saints' and All Souls' Days, which this year must have a deeper and more absolutely united meaning than they ever before have brought to the minds and consciences of Christian people throughout the world. Throughout the land, in the Old Country, and in many places in the Empire and abroad, in the homes of our American Brothers Across the Seas, and wherever Christ's Sacrifice for Humanity is recognised and understood, there must surely be a great awakening, resulting in a nearer and ever nearer approach to the Kingdom of Christ, recognition of the Fatherhood of Almighty God, the Sanctification of the Spirit, and reconcilement of man to his Maker through the sacrifice of Jesus Christ our Lord, through which and by which we may confidently continue our life on earth until we attain The Immortality our Saviour has won for us all.

When Stainer's anthem, "Who are These?" is sung in the cathedrals, in the Great Chapels attached to the Colleges of our Universities, wherever the Worship of Almighty God and the Immortality which awaits the faithful are understood and believed in, the recall of the Victory and the Glory and the Majesty and the Mercy of the Splendid Triumph which Our Dear Ones have reached by Entering on Immortality, must surely Awaken the Souls of Men and Women, Quicken their life-blood and intelligence, and on their knees make them render thanks to Almighty God, not only for the blessings of health and for the mercies they have enjoyed, but for the Assured Certainty of the Victory over Death, which awaits everyone of us who admit Christ to our hearts and renders ourselves up whole-heartedly and entirely to His Devoted Service for the Rest of Our Days. The most terrible trials, dangers, conflicts, doubts, hindrances, obstacles, and all that makes life on earth so difficult, are after all, when accepted by the faithful with Simple Faith in God's Mercy, but Sanctification by the Spirit and Salvation Through The Voluntary Sacrifice of Jesus Christ Who Despised the Cross and Endured the Shame for The Glory which Might Be Revealed. Certainly that Glory, its actuality, its magnificence and power must come home to every human being, who, in the simple-minded, earnest, purposeful devotion of the believer, yields up mind and spirit to the care of Almighty God, the Father, in The Christian's Full Assurance that in His Keeping all will be well here and hereafter, if we faint not but believe.

The writer has lived a long life and seen many days. It has been his privilege to be many times weary and overladen, to feel the joy and blessedness Shed through Simple Faith, and the Fatherly Care and Goodness of Almighty God. These in his experience have been never-failing from his earliest days to the present moment. On All Saints' and All Souls' Days in Victory Year he looks forward with a joyous confidence. Be the days yet to come weary, or be they long, the end will be Peace, through reconcilement with the Father by our Lord Jesus Christ and the sanctifying presence of the Holy Spirit which is ever renewed and always present with those who, when Christ knocks at the heart of His Servant, the Door opens that He may enter and remain with that Human Soul For Ever. So the worst evils that the world contained have felt the reality of the immortality of man. Our warriors have brought with them added strength in the conviction that the Eternal Arms are stretched over all those who believe and are content to Rest their Souls with God the Father and Christ Crucified, now and ever.

